# Control of Rta expression critically determines transcription of viral and cellular genes following gammaherpesvirus infection

**DOI:** 10.1099/vir.0.82548-0

**Published:** 2007-06

**Authors:** James R. Hair, Paul A. Lyons, Kenneth G. C. Smith, Stacey Efstathiou

**Affiliations:** 1 Cambridge Institute for Medical Research and the Department of Medicine, Wellcome Trust/MRC Building, University of Cambridge, Addenbrooke's Hospital, Cambridge CB2 2XY, UK; 2 Division of Virology, Department of Pathology, University of Cambridge, Tennis Court Road, Cambridge CB2 1QP, UK; 3 Juvenile Diabetes Foundation/Wellcome Trust Diabetes and Inflammation Laboratory, Cambridge Institute for Medical Research and the Department of Medicine, Wellcome Trust/MRC Building, University of Cambridge, Addenbrooke's Hospital, Cambridge CB2 2XY, UK

## Abstract

The replication and transcriptional activator (Rta), encoded by ORF50 of gammaherpesviruses, initiates the lytic cycle of gene expression; therefore understanding the impact of Rta on viral and cellular gene expression is key to elucidating the transcriptional events governing productive infection and reactivation from latency. To this end, the impact of altering Rta transcription on viral and cellular gene expression was studied in the context of a whole virus infection. Recombinant murine gammaherpesvirus (MHV)-68 engineered to overexpress Rta greatly accelerated expression of specific lytic cycle ORFs, but repressed transcription of the major latency gene, ORF73. Increased expression of Rta accelerated the dysregulation in transcription of specific cellular genes when compared with cells infected with wild-type and revertant viruses. A subset of cellular genes was dysregulated only in cells infected with Rta-overexpressing virus, and never in those infected with non-overexpressing viruses. These data highlight the critical role of Rta abundance in governing viral and cellular gene transcription, and demonstrate the importance of understanding how the relative expression of ORF50 during the virus life cycle impacts on these processes.

## INTRODUCTION

Gammaherpesviruses are lymphotropic viruses that establish lifelong latent infections, and are associated with the development of lymphoproliferative disorders. Murine gammaherpesvirus (MHV) 68 was isolated from a bank vole (Blaskovic *et al.*, 1980), and represents a genetically tractable system for the study of gammaherpesvirus pathogenesis (Nash *et al.*, 2001; Stevenson *et al.*, 2002).

Gammaherpesvirus immediate-early genes initiate viral lytic cycle gene expression, and thereby govern the switch between lytic and latent replication. Epstein–Barr virus (EBV) encodes two genes (BRLF1 and BZLF1) that transactivate the lytic cycle of replication (Schwarzmann *et al.*, 1998; Speck *et al.*, 1997). The gene product of BZLF1, Zta, is not well conserved among the gammaherpesviruses. BRLF1, which is well conserved and encodes the virus replication and transcriptional activator (Rta) protein, is homologous to the open reading frame (ORF) 50 of MHV68, Kaposi's sarcoma-associated herpesvirus (KSHV), rhesus rhadinovirus (RRV) and herpesvirus saimiri (HVS) (reviewed by West & Wood, 2003).

The Rta protein encoded by ORF50 transactivates viral gene expression, triggering the lytic replication cycle (Liu *et al.*, 2000; Nicholas *et al.*, 1991). KSHV Rta enhances its own transcription and transactivates expression of ORF57 (Deng *et al.*, 2000), properties which are also shared by HVS and MHV68 Rta (Liu *et al.*, 2000; Whitehouse *et al.*, 1998). MHV68 and RRV Rta can transactivate KSHV viral promoters, demonstrating that the murid and rhesus Rta proteins retain some functions of the human Rta (Damania *et al.*, 2004). In addition, MHV68 Rta transactivates the MHV68 ORF50, ORF57 and M3 promoters (Martinez-Guzman *et al.*, 2003; Pavlova *et al.*, 2005; Wu *et al.*, 2001), although other viral gene targets may exist. Studies in KSHV and MHV68 have demonstrated an obligate requirement for Rta expression to initiate lytic replication (Pavlova *et al.*, 2003; Zhu *et al.*, 2004). Moreover, expression of Rta alone is sufficient to disrupt latency and initiate lytic replication in KSHV, HVS and MHV68 (Goodwin *et al.*, 2001; Sun *et al.*, 1998; Wu *et al.*, 2000).

In addition to activating viral transcription, Rta has been shown to modulate the expression of cellular genes. Rta of KSHV activates transcription of human interleukin-6 (IL-6) (Deng *et al.*, 2002) and interacts with the signal transducer and activator of transcription (STAT) 3 protein, inducing transcription of STAT3-target genes (Gwack *et al.*, 2002). Recent work has highlighted a role for KSHV Rta in upregulating transcription of CD21 and CD23 in B cells (Chang *et al.*, 2005). These studies expressed Rta in isolation from other viral factors and the role of Rta in the alteration of cellular gene expression, when expressed in the context of viral infection, has yet to be studied.

We sought to elucidate how changes in the relative expression of Rta alter viral and cellular gene expression using an MHV68 engineered to overexpress Rta by using the murine cytomegalovirus (MCMV) IE1 promoter (termed M50; May *et al.*, 2004). In this way, we were able to study the transcriptional impact of altering Rta abundance when it was expressed in the context of virus infection. M50 infection strongly induced several viral genes that are putative targets for direct transactivation by Rta, but downregulated transcription of the major latent gene ORF73, indicating that lytic replication may actively repress latent gene expression. Specific cellular genes [including nuclear factor of *κ* light chain gene enhancer in B-cells inhibitor *α* (I*κ*B*α*) and nuclear hormone receptor Nur77] were dysregulated after M50 infection and also, later, during wild-type (WT)-MHV68 infection. Changes in the transcription of these common genes may be fundamental to the viral replicative cycle. A subset of genes displayed dysregulated transcription only when Rta was overexpressed, including genes involved in cell adhesion (VCAM-1), cell growth (FGF10) and inflammation (Tnfip6, CPA3). These findings highlight cellular genes of potential importance during MHV68 early lytic replication, and demonstrate the need to consider Rta abundance as a fundamental determinant of alterations to the cellular transcriptome early during lytic replication and reactivation from latency.

## METHODS

### Cell lines and virus infections.

NIH 3T3 cells were cultured in Dulbecco's modified Eagle's medium supplemented with fetal calf serum (FCS; 10 % v/v), 4 mM l-glutamine, 100 U penicillin G ml^−1^ and 100 mg streptomycin ml^−1^. Working stocks of virus were medium-purified from infected BHK21 cell supernatants by high-speed centrifugation (14 000 r.p.m. for 2 h in a Beckman Type 19 rotor) and stored at −70 °C. Infections were performed at an m.o.i. of 5 with WT-MHV68 (strain g2.4), Rta-overexpressing MHV68 (M50) or revertant virus (50R) (May *et al.*, 2004). For each viral infection, mock-infected (medium only) cells were included as controls.

### Microarray probe design and fabrication.

Custom mouse immunology oligo set arrays (immunoarrays) were obtained from the MRC Rosalind Franklin Centre for Genome Research microarray programme (Hinxton, UK). The immunoarray contains 50mer oligonucleotide probes representing 2434 mouse genes and expressed sequence tags (ESTs) (for details see Supplementary Table S1, available in JGV Online[Table t1]). To enable analysis of the MHV68 transcriptome, additional 50mer oligonucleotide probes representing 77 viral ORFs were incorporated into the immunoarray.

### RNA extraction and microarray hybridization.

RNA was extracted at 1 and 2 h post-infection (p.i.) from M50-, 50R- and WT-MHV68-infected 3T3 cells using RNeasy columns (Qiagen), according to the manufacturer's instructions. In a separate experiment, RNA was extracted at 1, 2, 4, 8 and 12 h p.i. from 3T3 cells infected with WT-MHV68. Contaminating DNA was removed by on-column digestion with 30 U DNase (Qiagen), and RNA concentration was determined using an ND-1000 spectrophotometer (NanoDrop Technologies). RNA (40 μg in 12.9 ml RNase-free H_2_O) was labelled with dCTP-Cy3 or dCTP-Cy5 (Amersham Biosciences) and hybridized to microarrays as previously described (Petalidis *et al.*, 2003). Hybridized microarrays were scanned using a microarray scanner (Agilent Technologies).

### Microarray data analysis.

Imagene software (BioDiscovery) was used at default settings to extract probe fluorescence intensities; poor data (flag=1) were discarded prior to analysis. For viral genes, background-subtracted fluorescence values were averaged for duplicate probes on each microarray, then normalized across all microarrays using the 75th percentile method (Dr Edward Wagner, University of California at Irvine, USA). Data from each time point were normalized separately.

For cellular genes, intensity-dependent normalization (LOWESS) was performed for all microarrays at each time point using GeneSpring software (Agilent Technologies). The reproducibility of the microarray data was confirmed to be consistently high for each experiment, with mean correlation coefficients for the WT time course and M50 experiments of 0.95 and 0.87, respectively (Supplementary Fig. S1, available in JGV Online). Viral genes dysregulated by Rta overexpression were those that were expressed (>100 fluorescence units) with a fluorescence value in M50-infected cells that was at least twofold different from that in WT-infected cells. Cellular genes dysregulated in the WT-MHV68 time course (1–12 h p.i.) were identified as up- or downregulated (infected/mock-infected) by >1.65×, *t*-test *P* value <0.05, and fluorescence in either infected or mock-infected Cy-dye channel >1000 units. Cellular genes dysregulated by Rta overexpression were identified as those changing in M50-infected cells (according to the criteria outlined previously) and not changing in WT- and 50R-infected cells. The 1.65-fold cut-off value represents an empirically determined 95 % confidence limit for calling differential expression based on ‘self vs self’ hybridization data (P. Lyons, unpublished data).

### Primer design and quantitative RT-PCR (Q-RT-PCR).

Primers for tumour necrosis factor-induced protein (Tnfip) 6, vascular cell adhesion molecule (VCAM)-l, fibroblast growth factor (FGF) 10, vascular endothelial growth factor (VEGF) and *β*-2 microglobulin (*β*2M; loading control) genes were designed using Primer3 (Supplementary Table S2, available in JGV Online) (Rozen & Skaletsky, 2000). RNA from infected cells at 2 h p.i. was reverse-transcribed using Super RT (HT Biotechnology), according to the manufacturer's instructions. RNA was extracted from three independent series of infections. Levels of Tnfip6, VCAM-1, FGF10 and VEGF were normalized to *β*2M loading controls by subtracting their *C*_t_ values from the *β*2M double antibody *C*_t_ value for that cDNA (ABI 7000 sequence detection system; Applied Biosystems; and SYBR Green; Qiagen). Levels of CPA3 were normalized to HPRT using Assay-On-Demand reagents (Applied Biosystems). Data analysis was performed using Excel (Microsoft).

### Western blotting.

Cell lysates were prepared from 3T3 cells infected for 10 h. Protein samples (50 mg) were subjected to SDS-PAGE, transferred to nitrocellulose membranes and incubated in 0.4 mg goat anti-mouse VCAM-1 ml^−1^ (R&D Systems) or an irrelevant primary antibody (0.4 mg goat anti-mouse IgM–biotin ml^−1^; Sigma-Aldrich). Membranes were washed in PBS/0.1 % Tween 20, incubated in secondary antibody (donkey anti-goat IgG–horseradish peroxidase; The Jackson Laboratory), washed in PBS/0.1 % Tween 20 and then in dH_2_O. Protein bands were visualized using ECL reagents (Amersham) and quantified using Quantity One software (Bio-Rad).

### FACS analysis.

Cells (5×10^5^) were infected for 10 h, washed three times in PBS/1 % FCS and stained using biotinylated goat anti-mouse VCAM-1 (2.5 mg ml^−1^; R&D Systems). Isotype controls were WT-infected cells stained with biotinylated goat anti-mouse IgM (2.5 mg ml^−1^; Sigma). Cells were washed, stained with streptavidin–FITC (1.25 mg ml^−1^; Amersham Biosciences), rewashed and analysed by flow cytometry (FACSCalibur; BD Biosciences). 7-Aminoactinomycin D (7AAD; Molecular Probes) was used as a marker of dead cells. Data analysis was performed using FCSPress (Ray Hicks, Department of Medicine, University of Cambridge, UK).

## RESULTS

### Accelerated lytic replication as a consequence of Rta overexpression leads to rapid changes in viral gene expression

**Fig. 1. f1:**
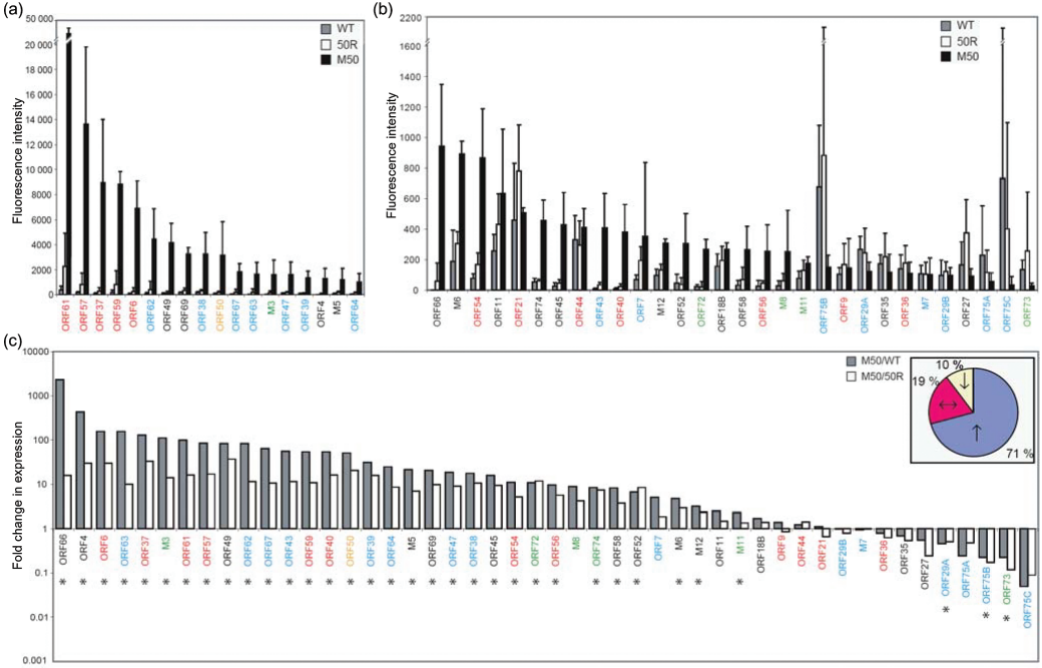
Viral gene expression 1 h after infection with WT-, 50R- and M50-MHV68. 3T3 cells were infected at an m.o.i. of 5. RNA was extracted and analysed by DNA microarray. Fluorescence intensity (relative expression) of high-expression (fluorescence >1000) MHV68 ORFs (a) and low-expression (fluorescence <1000) MHV68 ORFs (b) in infected cells. Bars show mean fluorescence intensities (*n*=3), ± sd. ORF colours: yellow, Rta; red, DNA replication/transactivation; blue, virion structural/transport/assembly protein; green, latency-associated; black, unknown/other function. (c) Histogram showing fold-change differences in ORF expression when M50-infected cells are compared to WT- (grey) or 50R- (white) infected cells. Statistically significant fold changes (when M50 was compared with WT; *P*<0.05 by Student's *t*-test) are indicated by asterisks. The pie chart shows percentage of ORFs that (relative to WT-MHV68-infected cells) were upregulated >2-fold (↑), unchanged (↔) or downregulated >2-fold (↓).

To address the impact of Rta abundance on viral gene transcription, 3T3 cells were infected with an MHV68 recombinant (M50) engineered to overexpress Rta (ORF50) as a consequence of placing Rta under control of the MCMV IE1 promoter (May *et al.*, 2004). As controls, WT- or M50 revertant (50R)-MHV68 infections were performed. Viral gene expression was determined at 1 and 2 h p.i. using a custom microarray carrying probes representing 77 MHV68 ORFs. Viral gene expression was low at 1 h p.i. in cells infected with WT- or 50R-MHV68 (Fig. 1a, b[Fig f1]). Increased Rta transcription profoundly upregulated expression of many ORFs, the most abundant being genes with roles in viral DNA replication, ORF61 [ribonucleotide reductase (RNR), large subunit], ORF57 (post-transcriptional regulator) and ORF37 (alkaline exonuclease) (Fig. 1a[Fig f1]).

To quantify these changes, the fold-change difference in expression of each ORF was calculated (Fig. 1c[Fig f1]). Overexpression of Rta upregulated the transcription of most MHV68 ORFs. Many of the most highly induced genes [including ORFs 6 (single-stranded DNA-binding protein), 37, 57 and 61] were also those that were abundantly expressed, indicating that increased Rta expression strongly induced transcription of these genes. Contrastingly, overexpression of Rta downregulated transcription of ORF73 (latency-associated nuclear antigen, LANA) and the three viral tegument/*N*-formylglycinamide ribotide amidotransferases (FGARAT; ORF75A, B and C). When the data were analysed to determine whether certain functional classes of viral genes (DNA replication/transactivation, virion structure/transport/assembly or latency-associated) were preferentially upregulated or downregulated, Rta overexpression was determined to affect all classes equally (*χ*^2^ analysis, data not shown).

At 2 h p.i., viral gene transcription in M50-infected cells remained greater than in WT- or 50R-infected cells (Supplementary Fig. S2a, b; available in JGV Online), but the fold-change differences were less striking than at 1 h p.i. (Supplementary Fig. S2c). Similar to the 1 h time point, overexpression of Rta reduced transcription of ORF73, ORFs 75A, B and C and affected all functional classes equally (data not shown).

Therefore, elevated transcription of Rta profoundly influenced viral gene transcription as early as 1 h p.i. Specific genes (including DNA replication ORFs 6, 37, 57 and 61) were strongly induced by elevated Rta levels, indicating that their induction via Rta may be fundamental to triggering lytic replication. Contrastingly, increased Rta suppressed ORF73 transcription, illustrating a mechanism by which the switch between latency and lytic replication may occur.

### Rta abundance is a fundamental determinant of cellular gene transcription

To determine the extent to which levels of Rta impact on cellular transcription, we identified genes whose transcription was altered in M50-infected cells, but not in WT- or 50R-infected cells at 1 and 2 h p.i., and compared these to transcriptional changes occurring in a separate WT infection time course.

#### Enhanced expression of Rta induces unique changes in cellular gene transcription and negatively regulates expression of tumour suppressor genes.

A striking finding of this study was that altering the expression of Rta had a profound influence on the cellular transcriptome (Table 1). M50 infection induced the transcriptional dysregulation of a cohort of 15 genes and one EST not observed to change in WT- or 50R-infected cells at the same 2 h p.i. time point, or in a separate WT infection time course (Table 1). Most of the M50-specific, upregulated genes encode secreted proteins, including carboxypeptidase (CP) A3, Tnfip6 and FGF10. Elevated expression of Rta downregulated the transcription of several tumour suppressor or pro-apoptotic genes [transforming growth factor *β*-induced transcript (TGFbli) 4, myeloid differentiation primary response gene (Myd) l18, I*κ*B*α*, CCAAT/enhancer binding protein (C/EBP) *β*, Kruppel-like factor (Klf) 4, CCCTC-binding factor (Ctcf) and Nur77] as well as members of the transforming growth factor (TGF) *β* signalling pathway [integrin *α* V (CD51), Myd118, TGFbli4 and dual specificity phosphatase (Dusp) l] mutations in which are associated with human malignancies. These data indicate that Rta abundance is a critical factor in determining the cellular transcriptional profile and suggest that the level of Rta expressed may influence cell survival.

#### Lower Rta expression targets dysregulation of cellular genes with roles in cell survival, adhesion, migration and mRNA processing.

The 29 genes and three ESTs differentially regulated at lower Rta expression in the WT infection time course were strikingly different from those dysregulated at higher Rta abundance (Table 1). The oncogenes Myc and ephrin receptor Epha2 were upregulated, whereas the pro-apoptotic zinc finger protein (Zfp) 36, Zfp36 C3H type-like (Zfp36l) 1, Klf2, inhibitor of DNA binding (Id) 3 and DNase II *α* (DNase2a) were downregulated. Jun, Jun proto-oncogene related gene d1 (JunD), Fos and Fosb were all downregulated and are components of the AP-1 complex that can exert either pro- or anti-apoptotic effects. Downregulation of Zfp36 and Zfp36l1 may additionally increase the stability of AU-rich element (ARE) domain-containing mRNAs (Blackshear, 2002). The expression of cell adhesion and migration-associated genes was also affected, with upregulation of the urokinase plasminogen receptor (uPAR), CD44 and small inducible cytokine (Scy) a2, but downregulation of Ly6 receptors, CD24a and integrin *β* (Itgb) 5. Therefore, lower Rta expression induced distinct transcriptional changes dominated by the dysregulation of genes implicated in cell survival, adhesion, migration and mRNA stability.

#### A conserved set of cellular genes are dysregulated at both high and low Rta abundance.

A subset of the cellular genes identified was dysregulated at both high (M50) and lower (WT) Rta abundance (Table 1). I*κ*B*α*, Dusp1 and Nur77 have roles in the negative regulation of inflammation, mitogen-activated protein kinase (MAPK) signalling and cell survival, respectively, whereas platelet-derived growth factor receptor *α* polypeptide (PDGFRA) is a potent activator of cell growth, survival and migration. The conservation of these transcriptional changes implies an important role in lytic replication and the potential biological relevance of these findings is outlined in the Discussion.

### Confirmation of microarray findings at the level of RNA and protein

**Fig. 2. f2:**
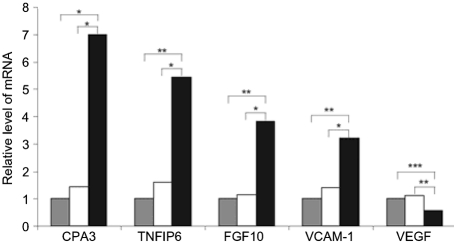
Q-RT-PCR confirmation of microarray data. 3T3 cells were infected (at an m.o.i. of 5) for 2 h with WT-, 50R- or M50-MHV68 and expression of CPA3, TNFIP6, FGF10, VCAM-1 and VEGF was determined using Q-RT-PCR. Bars show mean fold changes (*n*=3 infections) normalized to loading controls (*β*-2 microglobulin or glyceraldehyde-3-phosphate dehydrogenase) in cells infected with 50R- (white) and M50- (black) relative to WT-MHV68 (grey). *P* values according to Student's *t*-test are shown (*, *P*<0.05; **, *P*<0.01; ***, *P*<0.001). There was no statistically significant difference between WT- and 50R-MHV68 values for any of the genes tested.

We chose to study the link between Rta abundance and transcription further by RT-PCR, and examined a subset of cellular genes specifically dysregulated following M50 infection. Genes were chosen for their biological interest: CPA3 is the most highly upregulated transcript and encodes a carboxypeptidase that may function to degrade inflammatory leukotrienes (Reddanna *et al.*, 2003; Vendrell *et al.*, 2000); FGF10 is a member of a family of proteins with potent growth-promoting properties (Eswarakumar *et al.*, 2005); Tnfip6 suppresses the inflammatory response, which may facilitate viral growth, and has also been implicated in the regulation of lymphocyte migration and adherence (Milner & Day, 2003); VEGF is a pro-angiogenic factor, the expression of which is upregulated by EBV latent membrane protein 1 (LMP1) and KSHV ORF74 (Bais *et al.*, 1998; Murono *et al.*, 2001); VCAM-1 was selected despite having a *P* value >0.05, as its upregulation has been associated with several human herpesvirus infections (Allen *et al.*, 1996; Kim *et al.*, 2000; Pati *et al.*, 2001; Rahbar & Soderberg-Naucler, 2005). Q-RT-PCR confirmed the differential regulation of all of these genes (Fig. 2).

**Fig. 3. f3:**
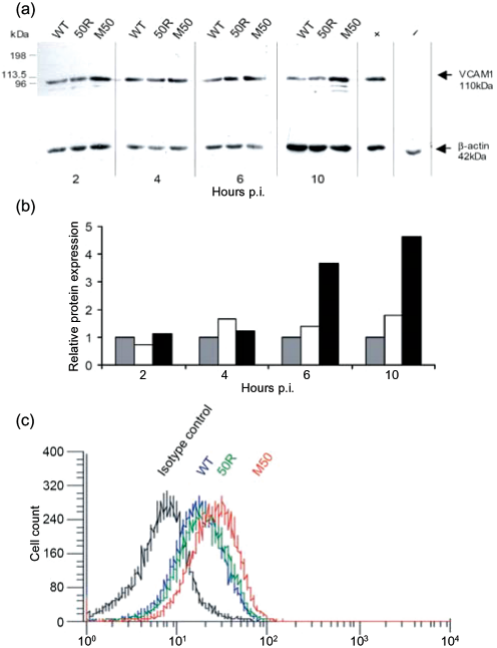
Expression of VCAM-1 protein in infected cells. 3T3 cells were infected with WT-, 50R- or M50-MHV68 for 2, 4, 6 and 10 h. (a) Cell lysates were subjected to gel electrophoresis, the proteins were transferred to a nitrocellulose membrane and VCAM-1 was detected using biotinylated anti-VCAM-1 with anti-IgG streptavidin–horseradish peroxidase antibodies. +, Uninfected 3T3 cells; −, uninfected 3T3 cells with irrelevant (anti-mouse IgM biotin) primary antibody. (b) Densitometry of the protein bands was performed and VCAM-1 values were normalized to *β*-actin controls. For each time point, VCAM-1 expression in 50R- (white) and M50- (black) infected cells is shown relative to that in WT-MHV68 (grey) -infected cells (set to 1). (c) FACS analysis of cell-surface VCAM-1 was performed using 3T3 cells infected for 10 h with WT- (blue), 50R- (green) or M50-MHV68 (red) and is shown relative to WT-MHV68-infected cells stained with isotype control antibody (black).

To test the correlation between levels of mRNA and protein, we chose to examine expression of VCAM-1 by Western blotting (Fig. 3a[Fig f3]). At 6 and 10 h p.i., M50 infection upregulated VCAM-1 expression 3.67- and 4.64-fold relative to that in WT- and 50R-infected cells, respectively (Fig. 3b[Fig f3]). The difference in expression of VCAM-1 between WT- and 50R-infected cells was never greater than 1.79-fold (10 h p.i.) at any time point tested, and was substantially lower than that induced by M50 infection. To confirm these findings using an independent measure of VCAM-1 abundance, FACS analysis of cells infected for 10 h with WT, 50R or M50 viruses was performed (Fig. 3c[Fig f3]). Expression of VCAM-1 in cells infected with WT- or 50R-MHV68 was almost identical, but in M50-infected cells it was 46 and 32 % greater than in WT- and 50R-infected cells, respectively. Therefore, increasing Rta expression in the context of virus infection upregulates VCAM-1 expression at the cell surface.

## DISCUSSION

The major aim of this study was to determine whether Rta abundance influences viral and cellular gene expression, since the regulation of Rta expression is likely to be cell-type dependent, and may differ between entry into lytic cycle following exogenous virus infection and reactivation from latency. Few studies have addressed the role of Rta in alteration of cellular gene transcription, and none have done this in the context of virus infection. To study the influence of Rta abundance on viral and cellular transcription in the context of virus infection, gene transcription in cells infected with MHV68 expressing Rta at normal (WT and 50R) or increased (M50) levels was compared. Elevated Rta expression induced the clearest differences in viral gene expression at 1 h p.i. These changes correlate with previous findings that increasing Rta expression accelerates virus replication (May *et al.*, 2004), although enhanced protein expression is probably restricted to early proteins as gene expression converges with that of WT and 50R viruses at later time points (Supplementary Fig. S2, available in JGV Online).

M50 infection reduced transcription of the latency gene ORF73 (LANA), extending previous findings that the ORF73 promoter is repressed by Rta (Coleman *et al.*, 2005), and offers a possible additional explanation for the latency deficit associated with M50 *in vivo* (May *et al.*, 2004). Splicing of MHV68 ORF73 occurs over the ORF75 locus (Coleman *et al.*, 2005), making it likely that the microarray probes against ORFs 75A, B and C cross-hybridize with unspliced ORF73 RNAs. It is therefore likely that the apparent downregulation of ORF75 transcription merely reflects the consequence of probe cross-hybridization.

Overall, these findings not only highlight new putative transactivation targets of Rta, but also demonstrate that Rta abundance negatively correlates with ORF73 transcription, and therefore potentially with latency.

The striking finding of this study with regard to changes in cellular gene expression is that altering the transcription of Rta has profound effects on the cell transcriptome. Lower expression of Rta during the WT-MHV68 infectious time course promotes a transcriptional profile seemingly favouring cell survival, with upregulation of anti-apoptotic Epha2 and Myc, and downregulation of many pro-apoptotic genes including Zfp36, Zfp36l1, Klf2 and Id3. The downregulation of the ARE-binding proteins (AUBP) Zfp36 and Zfp36l1, which bind and destabilize AU-rich mRNAs, including those encoding many proto-oncogene and cytokine RNAs, suggests that regulation of mRNA stability may also be important to the virus life cycle. This is in keeping with emerging evidence from other gammaherpesviruses such as KSHV, which encodes kaposin B, a protein involved in the stabilization of AU-rich cytokine transcripts (McCormick & Ganem, 2005). When Rta was expressed at a higher level, a subset of genes were identified that were transcriptionally altered only following M50 infection, but not following WT or 50R infection, suggesting that factors influencing Rta expression levels can profoundly influence cellular gene transcription. Our interest in studying the link between Rta abundance and cell function led us to confirm several M50-specific genes of interest by RT-PCR. Both VCAM-1 and Tnfip6 were upregulated, and are known to promote lymphocyte adherence (Milner & Day, 2003; Springer, 1995). VCAM-1 induction has been observed following herpes simplex virus and human cytomegalovirus infection (Kim *et al.*, 2000; Shahgasempour *et al.*, 1997). In addition, ORF74 of KSHV has been shown to positively regulate cell-surface VCAM-1 (Pati *et al.*, 2001). It may therefore be important to consider how cell adherence is affected during MHV68 infection as a product of Rta abundance.

Our interest in FGF10 and VEGFs was because these families of molecules are potent activators of cell growth, differentiation and migration. The finding that the transcription of growth factors such as FGF10 positively correlated with enhanced Rta expression demonstrates that the level of viral gene expression may impact on infected cells and also their surrounding tissues. Enhanced VEGF production is notable in human gammaherpesvirus-associated malignancies (Aoki & Tosato, 1999), although interestingly, increased MHV68 Rta expression reduced VEGF transcription. CPA3 is a poorly characterized enzyme (Reznik & Fricker, 2001), but it would be interesting to study whether its induction plays a role in the degradation of inflammatory leukotrienes, as reported for pancreatic carboxypeptidase (Reddanna *et al.*, 2003). Contrastingly, Tnfip6 is a well-documented, multifunctional protein with potent anti-inflammatory properties (Milner & Day, 2003). These data further highlight the important influence of Rta abundance on cellular transcription, and suggest that cell adherence, inflammation and growth factor release may be influenced by Rta expression.

Of significant interest was the identification of genes that were dysregulated both at elevated (M50) and lower (WT) levels of Rta. Such conserved changes may indicate genes whose dysregulation is fundamental to lytic replication. Nur77 is one such gene and encodes a pro-apoptotic protein whose function is suppressed by EBV nuclear antigen 2 (EBNA2) (Lee *et al.*, 2002). Another gene of potential importance is I*κ*B*α*, a negative regulator of nuclear factor *κ*B signalling that is important in antiviral inflammatory responses and the maintenance of latent gammaherpesvirus infections (Brown *et al.*, 2003; Hiscott *et al.*, 2001). It would be of great interest to study further whether subversion of apoptosis and inflammatory signalling via these molecules is fundamental to the early phases of MHV68 replication.

Whilst this study demonstrates the need to consider Rta abundance as a determinant of virus and cellular gene transcription, future work would seek to identify the mechanisms by which Rta abundance mediates these changes, and whether the effects are directly mediated by Rta or represent indirect effects as a consequence of accelerated lytic infection. For viral genes, this would include studying isolated promoter regions of putative Rta-responsive ORFs for their transcriptional response to Rta. The role of Rta abundance in altering cell adhesion and migration via VCAM-1 and Tnfip6 would be a significant area of interest, as would be studying the impact of cell-survival molecules such as Nur77 on virus replication. Ultimately, we would seek to identify how defined cellular transcriptional profiles correlate with Rta abundance in different infected cell types during primary infection and latency, and link these findings with alterations in cell function.

The findings in this study illustrate that alterations in Rta abundance during the earliest phases of lytic replication have profound influences on viral and cellular gene transcription. Rta abundance should therefore be considered as a potential factor in determining viral and cellular transcriptional changes occurring in discrete cell populations and at particular time points in the virus life cycle.

## Supplementary Material

[Supplementary Material]

## Figures and Tables

**Table 1. t1:** Cellular genes dysregulated after MHV68 infection Table shows fold changes for cellular genes that were dysregulated (criteria outlined in Methods) in infected cells. (a) Genes specifically dysregulated in M50-MHV68-infected cells at 2 h p.i. and (where applicable) also during a WT infection time course. No genes were identified as dysregulated in M50-infected cells at 1 h p.i. (b) Genes dysregulated only during a WT-MHV68 infection time course. For clarity, dysregulation of early growth response 1 at 1 h p.i. (−1.74) and the EST AW610815 at 1 and 2 h p.i. (1.79 and 1.74, respectively) are not shown. In all cases, positive and negative fold-changes indicate upregulation and downregulation of gene expression, respectively.

**Gene**	**Genbank accession nos**	**M50**	**WT**	**50R**	**WT**		
		**2 h p.i.**			**4 h p.i.**	**8 h p.i.**	**12 h p.i.**
(a)							
Carboxypeptidase A3, mast cell	NM_007753	35.25	*	*	*	*	*
Tumour necrosis factor-induced protein 6	U83903	3.66	*	*	*	*	*
Fibroblast growth factor 10	U94517	2.14	*	*	*	*	*
Vascular cell adhesion molecule 1†	M84487	1.65	*	*	*	*	*
Transforming growth factor *β*1-induced transcript 4	NM_009366	−1.66	*	*	*	*	*
EST expressed in B cells	AW495873	−1.69	*	*	*	*	*
Myeloid differentiation primary response gene 118	X54149	−1.70	*	*	*	*	*
Platelet-derived growth factor receptor *α*	M84607	−1.71	*	*	−1.67	*	−2.09
Nuclear factor I/A	D90173	−1.71	*	*	*	*	*
CDC-like kinase 3	AF033565	−1.71	*	*	*	*	*
Integrin alpha V (CD51)	U14135	−1.74	*	*	*	*	*
I*κ*B*α*	U57524	−1.74	*	*	*	*	−1.65
Vascular endothelial growth factor	U41383	−1.75	*	*	*	*	*
Dual specificity phosphatase 1	NM_013642	−1.83	*	*	*	−2.02	*
Enolase 2, *γ* neuronal	X52380	−1.83	*	*	*	*	*
Supressor of cytokine signalling 5	NM_019654	−1.88	*	*	*	*	*
CCAAT/enhancer binding protein (C/EBP) *β*	AY056052	−1.88	*	*	*	*	*
Kruppel-like factor 4 (gut)	U70662	−1.90	*	*	*	*	*
CCCTC-binding factor	U51037	−1.91	*	*	*	*	*
Nur77	NM_010444	−2.00	*	*	−2.20	−1.93	−1.74
(b)							
Urokinase plasminogen activator receptor	X62701	*	*	*	2.54	1.74	*
Small inducible cytokine A2	M19681	*	*	*	2.36	*	*
AW610703 (EST)	AW610703	*	*	*	2.18	*	*
CD44	M27130	*	*	*	1.97	*	*
Ephrin receptor A2	U07634	*	*	*	1.93	*	*
Myc	NM_010849	*	*	*	1.72	*	*
Peroxisome proliferator activator receptor *δ*	L28116	*	*	*	1.67	*	*
Jun oncogene	J04115	*	*	*	−1.65	*	*
Baculoviral IAP repeat-containing 2	U88909	*	*	*	−1.66	*	*
Viral envelope-like protein (G7e) gene	U69488	*	*	*	−1.66	*	*
Homeo box A3	Y11717	*	−1.65	−1.69	−1.73	*	*
Zinc finger protein 36, C3H type-like 1	M58566	*	*	*	−1.92	*	−2.00
Zinc finger protein 36	L42317	*	*	*	−2.27	−1.99	−2.48
Fos	NM_010234	*	*	*	−2.44	−2.25	−2.57
CD24a	X72910	*	*	*	*	−1.67	−2.14
Kruppel-like factor 2 (lung)	NM_008452	*	*	*	*	−1.68	−2.17
Jun proto-oncogene related gene d1	J04509	*	*	*	*	−1.76	*
Ly6 antigen	X04653	*	*	*	*	−1.88	−2.64
Fosb	NM_008036	*	*	*	*	−2.04	*
Early growth response 1	NM_007913	*	*	*	*	−2.07	*
AW824577 (EST)	AW824577	*	*	*	*	*	3.52
AW825364 (EST)	AW825364	*	*	*	*	*	−1.71
Inhibitor of DNA binding 3	NM_008321	*	*	*	*	*	−1.91
Deoxyribonuclease II *α*	AF045741	*	*	*	*	*	−1.92
Integrin beta 5	AF043256	*	*	*	*	*	−1.99
Platelet-derived growth factor receptor, *β*	X04367	*	*	*	*	*	−2.10
Lymphocyte antigen 6 complex, locus C	D86232	*	*	*	*	*	−2.63
Lymphocyte antigen 6 complex	M37707	*	*	*	*	*	−2.79

*Non-dysregulated genes that did not meet the following criteria; >1.65×, *t*-test *P* value <0.05 and fluorescence in either infected or mock-infected Cy-dye channel >1000 units. †*P*>0.05.
