# Supplementary material for: Control of Rta expression critically determines transcription of viral and cellular genes following gammaherpesvirus infection
Source: J Gen Virol. 2007 Jun;88(Pt 6):1689–97. doi: 10.1099/vir.0.82548-0 (PMC2884955; doi:10.1099/vir.0.82548-0)
Supplement: [Supplementary Material] [file supp_88_6_1689__index.html]

 Control of Rta expression critically determines transcription of viral and cellular genes following gammaherpesvirus infection -- Hair et al. 88 (6): 1689 Data Supplement - Supplementary Material -- Journal of General Virology

## 

### Control of Rta expression critically determines transcription of viral and cellular genes following gammaherpesvirus infection, by Hair, J. R., Lyons, P. A., Smith, K. G. C. and Efstathiou, S.

*Journal of General Virology* vol. **88**, part 6, pp. 1689–1697

**Supplementary Fig. S1.** Reproducibility of microarray data. [PDF] (71 KB)

**Supplementary Fig. S2.** Viral gene expression 2 h after infection with WT-, 50R- and M50-MHV68. [PDF] (803 KB)

**Supplementary Table S1.** Immunoarray raw data. [XLS] (1500 KB)

**Supplementary Table S2.** Q-RT-PCR primer sequences used in this study. [PDF] (62 KB)
