# Supplementary material for: Control of Rta expression critically determines transcription of viral and cellular genes following gammaherpesvirus infection
Source: J Gen Virol. 2007 Jun;88(Pt 6):1689–97. doi: 10.1099/vir.0.82548-0 (PMC2884955; doi:10.1099/vir.0.82548-0)
Supplement: [Supplementary Material] [file supp_88_6_1689__1.pdf]

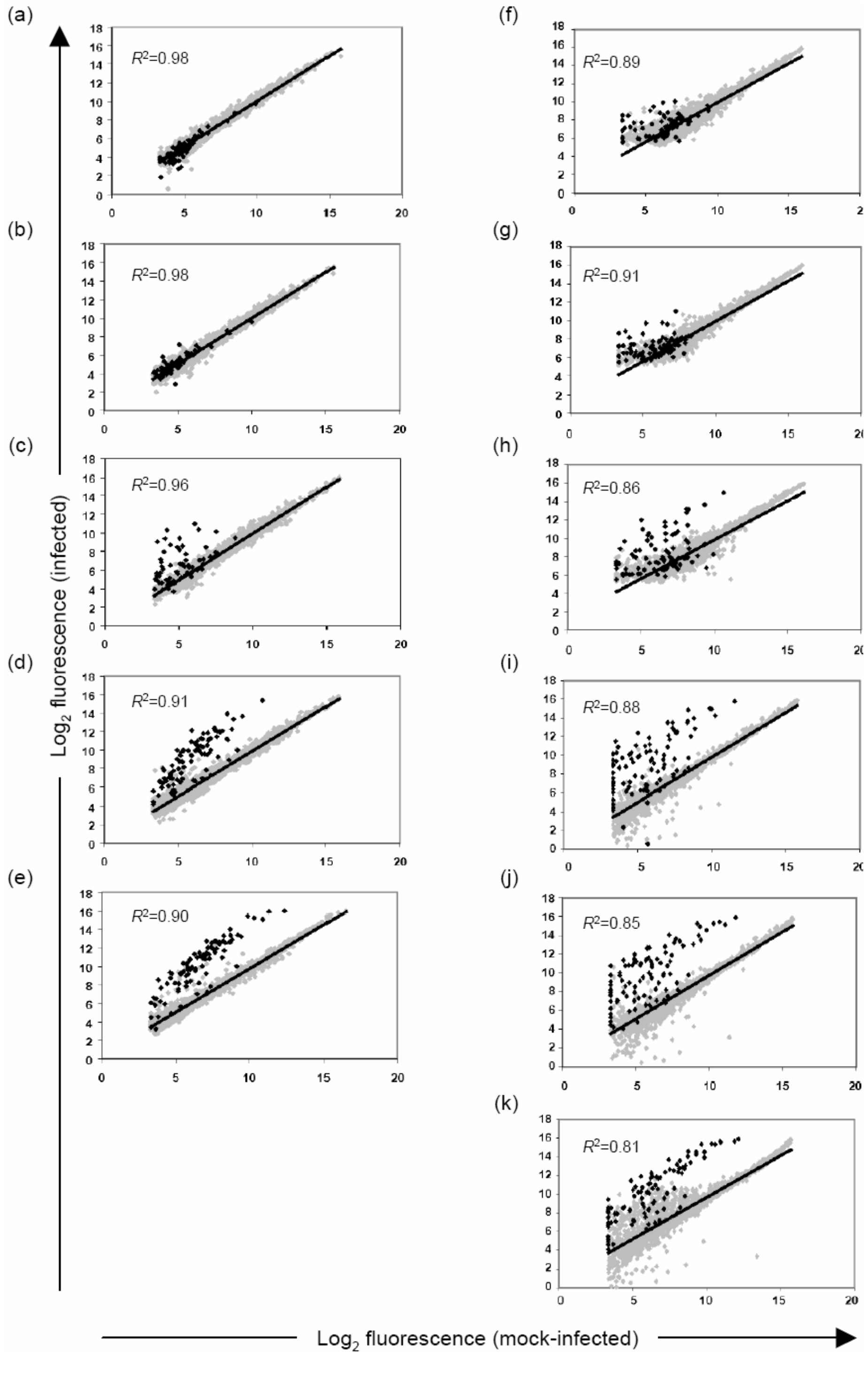

Supplementary Fig. S1. Reproducibility of microarray data. 3T3 cells were infected with WT-MHV68 and gene expression was compared with mock-infected cells at (a) 1 h p.i., (b) 2 h p.i., (c) 4 h p.i., (d) 8 h p.i. and (e) 12 h p.i. using microarrays (*n*=2). In a separate experiment, 3T3 cells were infected with WT- (f, i), 50R- (g, j) or M50-MHV68 (h, k) for 1 (f –h; *n*=3 or 4) or 2 (i–k; *n*=2) h, and gene expression was compared with that of mock-infected cells using microarrays. ◆, MHV68 probes; ◆, cellular gene probes.

Hair, J. R., Lyons, P., Smith, K. G. C. & Efstathiou, S. (2007). Control of Rta expression critically determines transcription of viral and cellular genes following gammaherpesvirus infection. *J Gen Virol* **88**, 1689–1697.
