# Supplementary material for: Control of Rta expression critically determines transcription of viral and cellular genes following gammaherpesvirus infection
Source: J Gen Virol. 2007 Jun;88(Pt 6):1689–97. doi: 10.1099/vir.0.82548-0 (PMC2884955; doi:10.1099/vir.0.82548-0)
Supplement: [Supplementary Material] [file supp_88_6_1689__2.pdf]

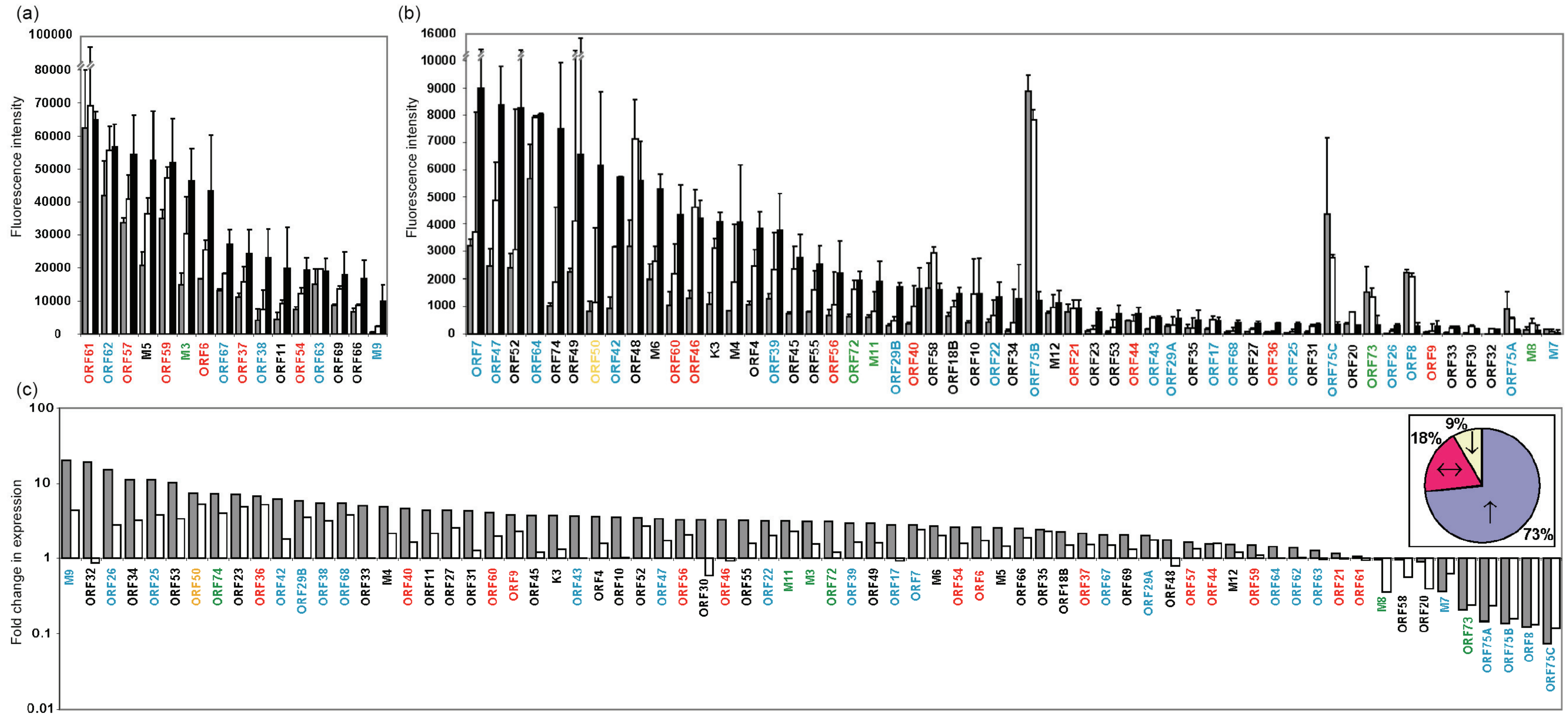

Supplementary Fig. S2. Viral gene expression 2 h after infection with WT-, 50R- and M50-MHV68. 3T3 cells were infected for 2 h with WT-, 50R- or M50-MHV68 (m.o.i.=5). RNA was extracted and analysed by DNA microarray. Fluorescence intensity (relative expression) of (a) high-expression (fluorescence >10 000) MHV68 ORFs and (b) low-expression (fluorescence <10 000) MHV68 ORFs in infected cells. Bars, mean fluorescence intensities (*n*=2) ±SEM. ORF colours: yellow, Rta; red, DNA replication/transactivation; blue, virion structural/ transport/assembly protein; green, latency-associated; black, unknown/other function. (c) Histogram showing fold-change differences in ORF expression when M50-infected cells are compared with WT- (grey) or 50R- (white) infected cells. Pie chart shows percentage of ORFs that (relative to WT-MHV68-infected cells) were upregulated >2-fold (↑), unchanged (↔) or downregulated >2-fold (↓).

Hair, J. R., Lyons, P., Smith, K. G. C. & Efstathiou, S. (2007). Control of Rta expression critically determines transcription of viral and cellular genes following gammaherpesvirus infection. *J Gen Virol* **88**, 1689–1697.
