# Supplementary material for: Control of Rta expression critically determines transcription of viral and cellular genes following gammaherpesvirus infection
Source: J Gen Virol. 2007 Jun;88(Pt 6):1689–97. doi: 10.1099/vir.0.82548-0 (PMC2884955; doi:10.1099/vir.0.82548-0)
Supplement: [Supplementary Material] [file supp_88_6_1689__3.pdf]

Supplementary Table S2. Q-RT-PCR primer sequences

Abbreviations: FGF, fibroblast growth factor; Tnfip, tumour necrosis factor-induced protein; VCAM, vascular cellular adhesion molecule; VEGF, vascular endothelial growth factor; β2M, β-2 microglobulin.

**Gene**	**GenBank accession no.**	**Primer**	**Sequence**
FGF10	U94517	Left	GCCACCAACTGCTCTTCTTC
		Right	GACGGCAACAACTCCGATT
Tnfip6	U83903	Left	CTCCGCCTACCAAGTGCTG
		Right	GGAAGTGCTTGAGCTATACAATG
VCAM-1	M84487	Left	AGTCTGGTGGAGGCACAG
		Right	CCAAGCAACACTCTCTGATTACG
CEGF	U41383	Left	CAGACTATCAGCGGACTCACC
		Right	GGAGTGAAGAACCAACCTCCT
β2M	NM_009735	Left	TGGTGCTTGTCTCACTGACC
		Right	AGTATGTTCGGCTTCCCATTC

Hair, J. R., Lyons, P., Smith, K. G. C. & Efstathiou, S. (2007). Control of Rta expression critically determines transcription of viral and cellular genes following gammaherpesvirus infection. *J Gen Virol* **88**, 1689–1697.
